# Modulation of Network Oscillatory Activity and GABAergic Synaptic Transmission by CB1 Cannabinoid Receptors in the Rat Medial Entorhinal Cortex

**DOI:** 10.1155/2008/808564

**Published:** 2008-12-01

**Authors:** Nicola H. Morgan, Ian M. Stanford, Gavin L. Woodhall

**Affiliations:** School of Life and Health Sciences, Aston University, Aston Triangle, Birmingham B4 7ET, UK

## Abstract

Cannabinoids modulate inhibitory GABAergic neurotransmission in many brain regions. Within the temporal lobe, cannabinoid receptors are highly expressed, and are located presynaptically at inhibitory terminals. Here, we have explored the role of type-1 cannabinoid receptors (CB1Rs) at the level of inhibitory synaptic currents and field-recorded network oscillations. We report that arachidonylcyclopropylamide (ACPA; 10 *μ*M), an agonist at CB1R, inhibits GABAergic synaptic transmission onto both superficial and deep medial entorhinal (mEC) neurones, but this has little effect on network oscillations in beta/gamma frequency bands. By contrast, the CB1R antagonist/inverse agonist LY320135 (500 nM), increased GABAergic synaptic activity and beta/gamma oscillatory activity in superficial mEC, was suppressed, whilst that in deep mEC was enhanced. These data indicate that cannabinoid-mediated effects on inhibitory synaptic activity may be constitutively active in vitro, and that modulation of CB1R activation using inverse agonists unmasks complex effects of CBR function on network activity.

## 1. INTRODUCTION

Cannabinoid receptors are a family of G-protein coupled, presynaptic receptors [1, 2]. Autoradiography studies using the cannabinoid receptor ligand CP55,940 [3–5] show that CB1Rs are distributed throughout neuronal tissue. These studies report a dense binding of CP55,940 in the basal ganglia, specifically the substantia nigra *pars reticulata* the globus pallidus (GP) and also in cerebellum. In the cerebrum, the hippocampal formation and the entorhinal cortex (EC) show the highest density of staining for CB1R.

Cannabinoids are known to exert powerful control over GABAergic inhibitory signalling in the CNS [6–8], and it is reported that CB1R inhibition of GABA_A_ receptor mediated synaptic transmission occurs through the inhibition of voltage-dependent calcium channels (VGCCs; [6]). In the hippocampus, the activation of presynaptic CB1R depresses GABA release onto postsynaptic target cells [9, 10], and in these studies, endogenous and exogenous CB1R agonists have been shown to reduce the amplitude and frequency of GABAergic spontaneous inhibitory postsynaptic currents (sIPSCs), but not to affect action-potential-independent miniature (m)IPSCs. Other studies have shown that cannabinoid receptor activation enhances network oscillatory activity [11]. However, in the parahippocampal region (PHR), the effects of cannabinoid receptors are less well described. Here, we have investigated the functional effects of CBRs on neuronal network activity modelled in vitro by kainate (KA-) induced persistent oscillations [8, 12]. Persistent oscillatory activity in the gamma frequency band (30–80 Hz) has been the most commonly reported and studied form of network activity in the in vitro slice preparation, and can be elicited by metabotropic glutamate receptors [13] or application of kainic acid [8, 12] and/or the muscarinic agonist carbachol Neuronal network oscillatory activity reflects the phasic inhibition of principal cells by GABAergic interneurones, which act to entrain and synchronize principal cell activity (Cobb et al., [14]). The mEC has been reported to express gamma oscillations (30–100 Hz) in response to application of nanomolar concentrations of kainate [15, 16], and oscillatory power was greatest in superficial layers II/III [15].

## 2. MATERIALS AND METHODS

Combined EC-hippocampal slices were prepared from young male Wistar rats (50–110 g) as previously described [17]. All experiments were performed in accordance with the UK Animals (Scientific Procedures) Act 1986 and European Communities Council Directive 1986 (86/609/EEC). Rats were anaesthetised with isoflurane and N_2_/O_2_, until cardiorespiratory arrest, and decapitated. The brain was rapidly removed and immersed in oxygenated artificial cerebrospinal fluid (ACSF) chilled to 4°C. Slices (450 *μ*m) were cut using a vibrating microtome (MicroM, Germany), and stored in ACSF continuously bubbled with 95% O_2_/5% CO_2_, at room temperature. Following a recovery period of at least 1 hour, individual slices were transferred to a recording chamber mounted on the stage of an Olympus (BX50WI) upright microscope. The chamber was continuously perfused with oxygenated ACSF at 30–32°C, at a flow rate of approximately 2 mL/min. The ACSF contained the following (in mM): NaCl (126), KCl (3.25), NaH_2_PO_4_ (1.25), NaHCO_3_ (24), MgSO_4_ (2), CaCl_2_ (2.5), and D-glucose (10). The solution was continuously bubbled with 95% O_2_/5% CO_2_ to maintain a pH of 7.4. Neurones were visualized using differential interference contrast optics and an infrared video camera.

Patch-clamp electrodes were pulled from borosilicate glass (1.2 mm OD, 0.69 ID; Harvard Apparatus) and had open tip resistances of 4-5 MΩ. They were filled with a solution containing the following (in mM): CsCl (90), HEPES (33), QX-314 (5), EGTA (0.6), MgCl_2_ (5.0), TEA-Cl (10), phosophocreatine (7) ATP (4), GTP (0.4). The solution was adjusted to 290 mOsmol with sucrose and to pH 7.4 with CsOH. Whole-cell voltage clamp recordings were made from neurones in layers II and V of the medial division of the EC, using an Axopatch 700 series amplifier (Molecular Devices, USA). The holding potential in all cases was –70 mV. Under these experimental conditions, layer II/V neurones exhibited sIPSCs, mediated by GABA acting primarily at GABA_A_ receptors.

Data were recorded directly to computer hard disk using AxoScope software (Molecular Devices, USA). Mini Analysis (Synaptosoft, USA) was used for analysis of sIPSCs offline. sIPSCs were detected automatically using a threshold-crossing algorithm, and their frequency and amplitude are analysed. 200 sIPSCs were sampled during a continuous recording period for each neurone under each condition. The nonparametric Kolmogorov-Smirnoff (KS) test was used to assess the significance of shifts in cumulative probability distributions of interevent interval (IEI). Differences between drug and control situations in studies of sIPSCs were assessed by means of a one-way ANOVA. All error values stated in the text refer to the S.E.M.

All salts used in preparation of ACSF were Analar grade and purchased from Merck/BDH (UK). LY320135 and ACPA were obtained from Tocris Cookson (UK).

For field recordings of oscillatory activity, slices were placed into an interface chamber (BRSC-1, Digitimer, UK) and the chamber was continuously perfused with oxygenated ACSF at 30–32°C, at a flow rate of approximately 1-2 mL/min. Extracellular population recordings were made with glass microelectrodes filled with aCSF, of resistance 1–3 MΩ. Signals were amplified 1000-fold and recorded unfiltered. Low amplitude 50 Hz interference was removed using a HumBug (Quest Scientific, Canada). Signals were digitized and recorded at 10 kHz using an NPI EXT-02F amplifier (NPI, Germany) and pClamp 10 software (Molecular Devices, USA). Following 30–90 minutes control period of stable oscillatory activity, drugs were applied to the bath in known concentration. Pharmacological oscillatory activity was analysed using the fast Fourier transform (FFT, Clampfit 10), cross-correlation analysis and Morlet-wavelet time-frequency spectrogram analysis (MatLab 2007R, Mathworks). Student *t*-tests were carried out to determine statistical significance.

We analysed oscillations at beta (15–29 Hz) and gamma (30–90 Hz) bands, using bandpass filters (Clampfit 10.1) and measurement of the area under the power spectrum curve in Sigmaplot 8.0.

## 3. RESULTS

**Figure 1:**
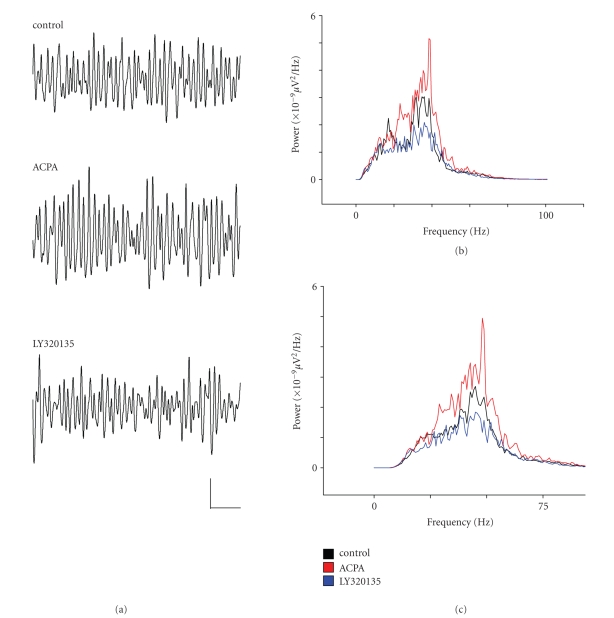
The effects of ACPA and LY320135 on *γ*-band activity in mEC layer II. (a) Example traces from layer II showing *γ*-oscillations under conditions in which ACPA (10 *μ*M) or LY320135 (500 nM) were applied. (b) Plot of power spectral density during drug application (filtered between 2–100 Hz). Control (black line), ACPA (red line), LY320135 (blue line). (c) Similar plot to (b) bandpass filtered between 30–90 Hz. Scale bar = 200 milliseconds × 100 *μ*V.

Whilst recording from layers II and V of the mEC, we applied the CBR agonist arachidonylcyclopropylamide (ACPA) at 10 *μ*M, onto slices from which stable gamma activity had been induced by 300–400 nM kainate. As Figure 1(a) shows, KA-induced gamma oscillations in layer II were broadly similar to those reported by Cunningham et al. [15]. Hence, mean area power in the *γ*-band was 561 ± 179 *μ*V^2^ and mean control gamma frequency was 40.7 ± 2.4 Hz. Following 40–60 minutes bath application of ACPA, we applied the CB1R-specific inverse agonist LY320135 (500 nM; [18]). Figure 1(b) shows the power spectral density of activity bandpass filtered between 2–100 Hz, and Figure 1(c) shows similar data filtered at gamma frequency (30–90 Hz). As Figure 1(c) shows, there was a tendency towards an increase in gamma power in ACPA in some recordings, but this was not significant overall (*P* ≥ .19, *n* = 9). In pooled data, ACPA did significantly reduce mean peak gamma frequency to 35.6 ± 1.8 Hz (*P* ≤ .04, *n* = 9), although this effect was variable, and some recordings showed multiple peaks.

Following perfusion of the CB1R-specific inverse agonist LY320135, there was a marked reduction in normalised gamma power to 39.4 ± 10.1% of control, and this was highly significant (*P* ≤ .0006, *n* = 9). In addition, mean peak frequency returned to 41.2 ± 1.8 Hz (*P* ≤ .04, *n* = 9).

**Figure 2:**
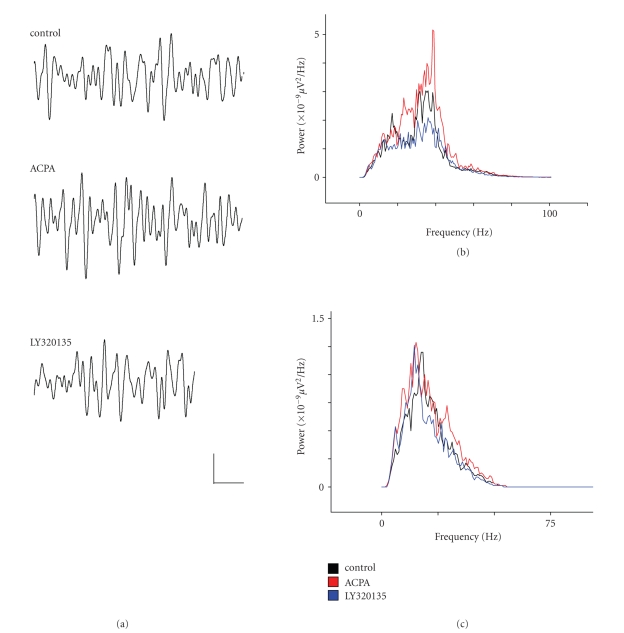
The effects of ACPA and LY320135 on *β*-band activity in mEC layer II. (a) Example traces from layer II showing *β*-oscillations under conditions in which ACPA (10 *μ*M) or LY320135 (500 nM) were applied. (b) Plot of power spectral density during drug application (filtered between 2–100 Hz). Control (black line), ACPA (red line), LY320135 (blue line). (c) Similar plot to (b) bandpass filtered between 15–29 Hz. Scale bar = 200 milliseconds × 50 *μ*V.

When beta power in layer II was measured, we noted a similar pattern of drug responses to that observed for gamma activity. Mean area power in the beta band was lower than that of gamma activity at 26 ± 6 *μ*V^2^ and mean peak beta frequency in control conditions was 25.6 ± 1.4 Hz. Figure 2(a) shows the power spectral density of activity bandpass filtered between 2–100 Hz, and Figure 2(c) shows similar data filtered at beta frequency (15–29 Hz). As Figure 2(c) shows, there was no significant change in beta power in ACPA (81.4 ± 15% of control, *P* ≥ .14, *n* = 9), and ACPA had no significant effect on mean peak beta frequency (27.6 ± 1.43 Hz, *P* ≥ .25, *n* = 9). However, when we added the CB1R-specific inverse agonist LY320135, there was a reduction in normalised beta power to 57 ± 13% of control, and this was highly significant (*P* ≤ .008, *n* = 9). LY320135 had no effect on mean peak frequency (27.9 ± 0.52 Hz, *P* ≥ .4, *n* = 9).

During the above experiments, we simultaneously recorded oscillatory activity in deep entorhinal cortex (layer V). Oscillatory activity in layer V was lower in power in layer V compared to layer II, with mean area gamma power just 60 ± 10 *μ*V^2^ and mean peak frequency was similar to layer II at 39.19 ± 3.1 Hz.

**Figure 3:**
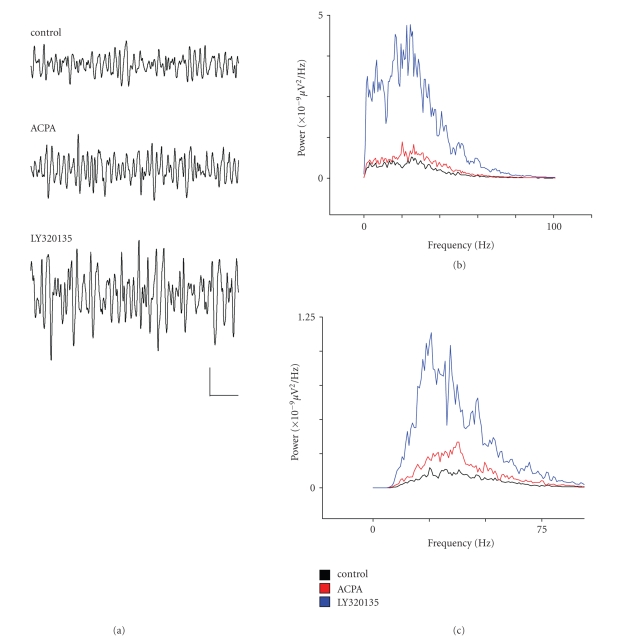
The effects of ACPA and LY320135 on *γ*-band activity in mEC layer V. (a) Example traces from layer V showing *γ*-oscillations under conditions in which ACPA (10 *μ*M) or LY320135 (500 nM) was applied. (b) Plot of power spectral density during drug application (filtered between 2–100 Hz). Control (black line), ACPA (red line), or LY320135 (blue line). (c) Similar plot to (b) bandpass filtered between 30–90 Hz. Scale bar = 200 milliseconds × 50 *μ*V.

When we applied ACPA whilst recording in layer V we observed a significant increase in mean gamma power (Figure 3(a)), by 38.1 ± 13.4% of control (*P* ≤ .03, *n* = 9), however, baseline gamma power was very low in this layer, and the absolute change in gamma power was difficult to discern. Peak frequency was again slightly reduced to 36.0 ± 2.4 Hz, but this was not significant (*P* ≥ .31, *n* = 9). On subsequent addition of the CB1R-specific inverse agonist LY320135, there was a strong increase in normalised gamma power to 108.4 ± 58% of control, and this reached significance (*P* = .049, *n* = 9). Again, LY320135 did not significantly alter mean peak gamma frequency (35.7 ± 2.41 Hz in LY320135, *P* ≥ .45, *n* = 9).

**Figure 4:**
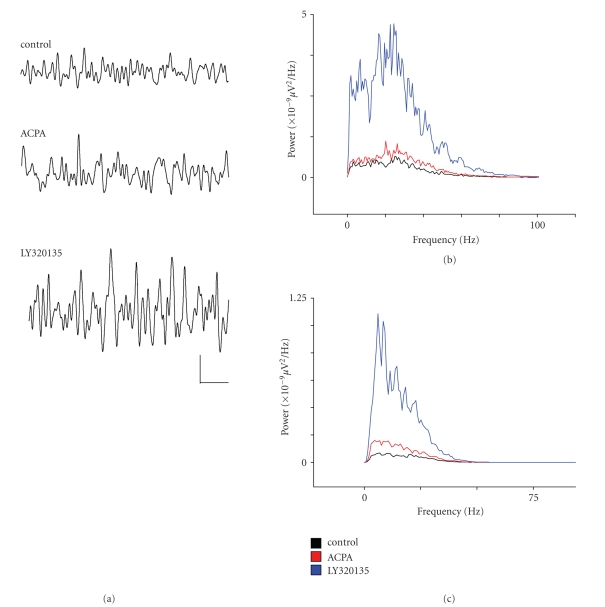
The effects of ACPA and LY320135 on *β*-band activity in mEC layer V. (a) Example traces from layer V showing *β*-oscillations under conditions in which ACPA (10 *μ*M) or LY320135 (500 nM) were applied. (b) Plot of power spectral density during drug application (filtered between 2–100 Hz). Control (black line), ACPA (red line), LY320135 (blue line). (c) Similar plot to (b) bandpass filtered between 15–29 Hz. Scale bar = 200 milliseconds × 50 *μ*V.

When beta power in layer V was analysed, we noted a similar pattern of drug responses to that observed for gamma activity. Mean area power in the beta band was lower than that of gamma activity at 9.6 ± 0.6 *μ*V^2^ and mean control beta frequency was 27.9 ± 0.52 Hz. Figure 4(a) shows field oscillations recorded in layer V before drug application and during ACPA and LY320135 periods. Figure 4(b) shows the power spectral density of activity bandpass filtered between 2–100 Hz, and Figure 4(c) shows similar data filtered at beta frequency (15–29 Hz). As Figure 4(c) shows, there was a slight tendency towards an increase in beta power (by 27 ± 14%) in ACPA in some recordings, but this was not significant overall (*P* ≥ .06, *n* = 9). ACPA had no significant effect on mean peak beta frequency (28.4 ± 0.7 Hz, *P* ≥ .5, *n* = 9). When we next applied the inverse agonist LY320135, we noted an increase in normalised beta power by 142.4 ± 88% of control, and this just failed to reach significance (*P* ≤ .07, *n* = 9). LY320135 (26.3 ± 1.5 Hz, *P* ≥ .3, *n* = 9) had no effect on peak frequency.

We hypothesised that the lack of effects of ACPA in layer II might reflect constitutive or tonic activation of CBR, perhaps due to persistent kainate-induced activation of pyramidal neurones. To test this hypothesis, we applied LY320135 in the absence of ACPA. Application of LY320135 suppressed gamma band activity to 19.5 ± 11% of control, and this was highly significant (*P* ≤ .01, *n* = 5). When beta activity was measured, it was apparent that in LY320135, there was a significant reduction in mean normalised beta power (58.4 ± 12% of control; *P* ≤ .04, *n* = 5).

**Figure 5:**
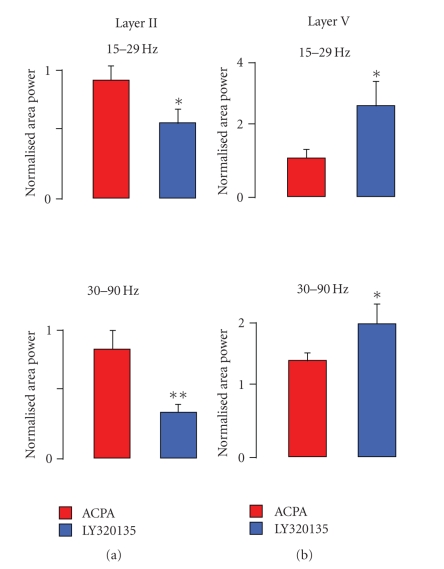
Summary of the effects of cannabinoid ligands on oscillatory activity in mEC. (a) Bar charts showing the effects of cannabinoid ligands in layer II on normalised area power at *γ* and *β* frequencies. (b) Bar charts showing the effects of inverse agonists alone in layer V on normalised area power at *γ* and *β* frequencies.

The data presented up to this point indicated that, in general, gamma and beta power decreased in layer II in response to blockade or inverse agonism of CB1R, and that in layer V, the opposite was seen, with an increase in gamma and beta power. Figures 5(a)-5(b) shows summary bar charts indicating the effects of ACPA and LY320135 on normalised mean area power in the gamma and beta bands in layers II and V of the mEC.

We hypothesised that the alterations in oscillatory power seen during drug application would relate to the effects of ACPA and LY320135 on sIPSCs impinging on neurones in deep and superficial entorhinal cortex. To measure these effects, we performed whole-cell voltage clamp recordings of sIPSCs, whilst bath applying ACPA and LY320135 at concentrations similar to those used above.

## 4. ACPA AND LY320135 HAVE SUBTLE EFFECTS ON sIPSC AMPLITUDE AND FREQUENCY IN mEC LAYER II

**Figure 6:**
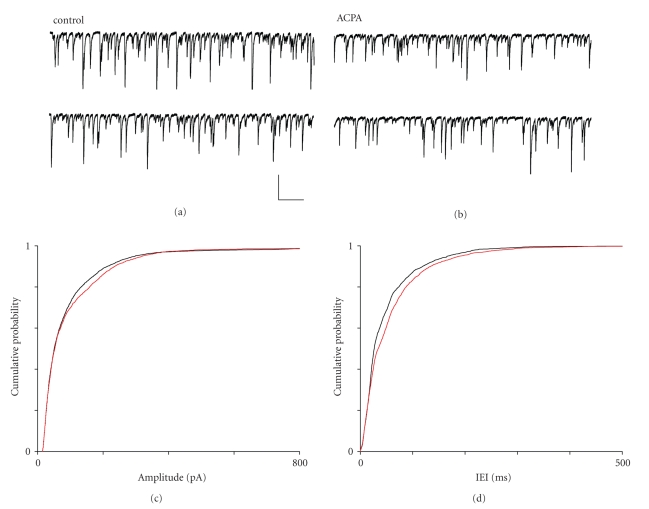
The effects of ACPA on sIPSCs in mEC layer II. (a) Recording from a layer II neurone under control conditions and (b) in the presence of ACPA (10 *μ*M). (c) Cumulative probability plot for sIPSC amplitude under control (black) and ACPA (red) conditions. (d) Cumulative probability plot for sIPSC IEI under control (black) and ACPA (red) conditions. Scale bars 500 millisconds × 100 pA.

**Figure 7:**
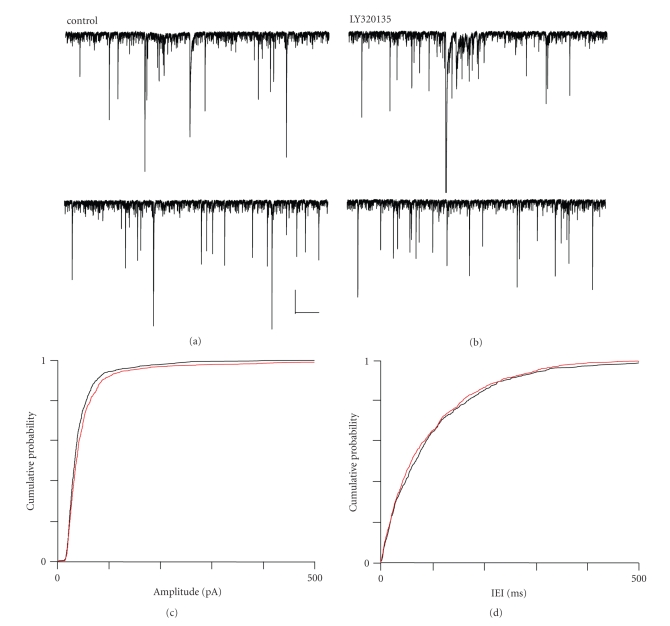
The effects of LY320135 on sIPSCs in mEC layer II. (a) Recording from a layer II neurone under control conditions and (b) in the presence of LY320135 (500 nM). (c) Cumulative probability plot for sIPSC amplitude under control (black) and LY320135 (red) conditions. (d) Cumulative probability plot for sIPSC IEI under control (black) and LY320135 (red) conditions. Scale bars 500 milliseconds × 250 pA.

Figure 6(a) shows typical recordings of inward sIPSCs made from a layer II pyramidal neurone. As Figure 6(b) shows, the application of ACPA (10 *μ*M) had subtle effects on sIPSCs in layer II, decreasing their frequency without affecting mean amplitude. Cumulative probability plots for sIPSC amplitude in the presence of ACPA (Figure 6(c)) indicated a shift in amplitude distribution, and mean amplitude showed a slight increase from 101.7 ± 3.2 pA in control to 108.3 ± 3.4 pA in ACPA, but this was nonsignificant (*P* ≥ .168, ANOVA). When we analysed amplitude distribution using the nonparametric Kolmogorov-Smirnov test, the shift towards higher amplitude sIPSCs was just significant (*P* ≤ .021 KS test). In the case of interevent interval (IEI; the reciprocal of frequency), we noted a shift to the right in the cumulative probability plot (Figure 6(d)), indicating an increase in the likelihood of greater IEI values (reduced frequency). Mean median IEI increased from 34.6 ± 5.5 milliseconds in control to 47.6 ± 9.7 milliseconds in ACPA (*P* ≤ .0001, ANOVA, *n* = 5), and the increase in IEI time distribution towards higher values was highly significant (*P* ≤ .006, KS test). When we performed similar experiments using LY320135 (500 nM), we noted effects which tended towards the opposite of those seen with ACPA, that is, increased sIPSC amplitude and frequency. Figure 7(a) shows a typical recording of inward sIPSCs made from a layer-II pyramidal neurone. As Figure 7(b) shows, the application of LY320135 increased sIPSC frequency and amplitude. Mean amplitude increased from 51.7 ± 3.6 pA in control to 69.0 ± 5.9 pA in LY320135, and this was significant (*P* ≤ .013, ANOVA, *n* = 6). Similarly, the shift in distribution towards larger amplitudes was highly significant (*P* ≤ .002, KS test). The mean median IEI showed a slight decrease from 86.6 ± 15.8 milliseconds in control to 80.6 ± 16.1 milliseconds LY320135, but this decrease in IEI time was not significant (*P* ≥ .116, ANOVA, *n* = 6).

The cumulative probability plots for sIPSC amplitude (Figure 7(c)) and IEI (Figure 7(d)) in the presence of LY320135 indicate the shifts in distribution of these parameters in the presence of LY320135.

## 5. ACPA AND LY320135 HAVE MARKED EFFECTS ON sIPSC AMPLITUDE AND FREQUENCY IN mEC LAYER V

**Figure 8:**
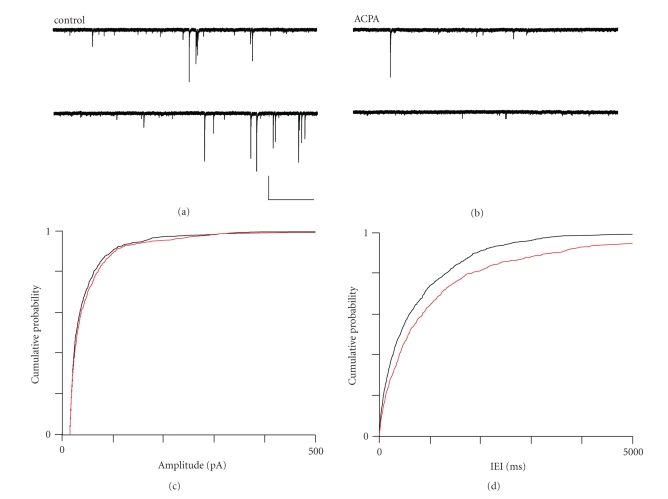
The effects of ACPA on sIPSCs in mEC layer V. (a) Recording from a layer V neurone under control conditions and (b) in the presence of ACPA (500 nM). (c) Cumulative probability plot for sIPSC amplitude under control (black) and ACPA (red) conditions. (d) Cumulative probability plot for sIPSC IEI under control (black) and ACPA (red) conditions. Scale bars 2000 milliseconds × 250 pA.

In contrast to the effects observed in layer II and layer V we noted a significant reduction in sIPSC frequency in response to ACPA application. As Figure 8(a) shows, sIPSCs in layer V are considerably less frequent than those in layer II (see Woodhall et al., [17]). When ACPA was applied, sIPSC frequency was greatly attenuated (Figure 8(b)), but there was no overall shift in amplitude distribution (confirmed by a nonsignificant KS test (*P* ≥ .23)). Mean amplitude rose slightly from 59.41 ± 7.33 pA in control to 70.49 ± 8.89 pA in ACPA, but this increase was not significant (*P* ≥ .33, ANOVA, *n* = 6). When we analysed IEI, the change in distribution towards larger IEI values was significant (*P* ≤ .0001 KS test), and mean median IEI was found to increase very significantly from 792 ± 41 milliseconds in control to 1317 ± 75 milliseconds in ACPA (*P* ≤ .0001, ANOVA, *n* = 6). This effect of ACPA on IEI in layer V was consistent in all recordings. Cumulative probability plots for sIPSC amplitude (Figure 8(c)) and IEI (Figure 8(d)) illustrate the effects of ACPA on sIPSC amplitude and IEI.

**Figure 9:**
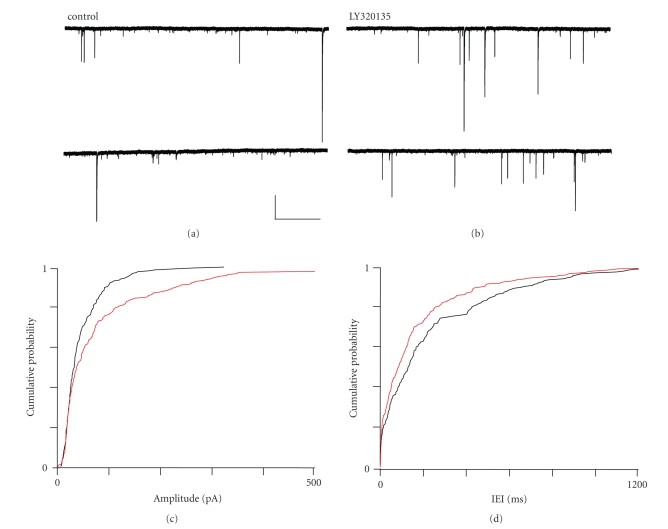
The effects of LY320135 on sIPSCs in mEC layer V. (a) Recording from a layer V neurone under control conditions and (b) in the presence of LY320135 (500 nM). (c) Cumulative probability plot for sIPSC amplitude under control (black) and LY320135 (red) conditions. (d) Cumulative probability plot for sIPSC IEI under control (black) and LY320135 (red) conditions. Scale bars 2000 milliseconds × 250 pA.

When we performed similar experiments using LY320135 (500 nM), we again noted robust effects, which opposed those seen with ACPA, that is, increased sIPSC amplitude and frequency. Figure 9(a) shows a typical recording of inward sIPSCs made from a layer-II pyramidal neurone. As Figure 9(b) shows, the application of LY320135 had marked effects on sIPSCs in layer V, increasing their frequency and amplitude. Mean sIPSC amplitude in layer V increased from 47.0 pA ± 3.0 to 87.9 pA ± 7.4 pA in LY320135, and this increase was significant (*P* ≤ .001, ANOVA *n* = 7). Cumulative probability plots for sIPSC amplitude in the presence of LY320135 (Figure 9(c)) show the shift in amplitude distribution, and this was confirmed statistically (*P* ≤ .0004, KS test). In the case of IEI, we noted a shift to the left in the cumulative probability plot (Figure 9(d)), indicating an increase in the likelihood of lower IEI values (increased frequency). During LY320135 application the mean median IEI decreased from 477.1 ± 108.0 milliseconds in control to 300.0 ± 71.5 milliseconds in LY320135 showing that an overall increase in sIPSC frequency has occurred. The decrease in mean median IEI between control and LY320135 periods was significant (*P* ≤ .014 ANOVA, *n* = 7), as was the change in distribution (*P* ≤ .016, KS test).

## 6. DISCUSSION

We found that the cannabinoid receptor agonist ACPA had little effect on either oscillatory activity or synaptic inhibition in superficial layers of the mEC, and that effects in deep layers were more robust, especially in the case of sIPSC frequency. However, the inverse agonist, LY320135, strongly suppressed oscillatory activity in superficial mEC even while its effects on sIPSC frequency and amplitude were not great. We also observed that suppression of oscillatory activity in layer II by LY3201235 was accompanied by augmentation of oscillatory power in layer V, and that the suppression of sIPSCs by ACPA and subsequent enhancement by LY320135 in this layer were marked.

We have reported previously [17] that spontaneous inhibition is much greater in superficial layer II of mEC than in deep layer V. In addition, more than 90% of IPSCs in layer II are action-potential (AP) independent, whereas in layer V, AP-dependent events comprise a much greater (>50%) proportion of sIPSCs. Given that CBRs act only on Ca^2+^ dependent release of GABA and have no effect on mIPSCs ([8]; it seems likely that cannabinoid ligands would show greater effects in the deep layers, where activity is low at baseline, and probably more sensitive to modulation since it is more likely to be AP-dependent.

Since, compared to layer II, both ACPA and LY320135 had more profound effects on synaptic inhibition in layer V, it seems likely that the relative dominance of mIPSCs in layer II may mask CB1R effects on the minority of AP-dependent sIPSCs to some degree. The lack of a robust effect of ACPA on oscillatory activity in layer V suggests, however, that CB1R may already be activated by ongoing network activity, and that further attempts at activation using an agonist did not increase any effect that CB1R might have on oscillatory power. This appears to be supported by the effects of the inverse agonist LY320135 in layer II. Here, we observed a robust reduction in both beta and gamma power in layer II, suggesting that CB1R do contribute to maintaining oscillatory activity in this layer. The apparently contradictory result of enhanced oscillatory activity in layer V in response to LY320125 may relate to effects that are secondary to activity in layer II, for example, Bragin [11], working in vivo, noted that ablation of superficial EC causes augmented oscillatory activity in CA3-CA1, and it may be that a similar mechanism allows suppression of oscillations in layer II to unmask activity in layer V, which receives inputs from CA1. Similarly, previous reports [15] indicate that superficial layers (especially layer III) show the strongest gamma power, perhaps suggesting a role in driving oscillatory activity in other layers. However, layer V is not driven directly by layers II or III [19], and hence any effect in layer V may well be indirect.

Cannabinoid receptors exert powerful control over GABA release from presynaptic terminals, with CB1 receptors having been shown to suppress both IPSPs and IPSCs in pyramidal neurones (IPSPs, [20]; IPSCs, [8]). Endocannabinoids, such as 2-arachidonyl glycerol and anandamide also suppress inhibition in CNS (see [21], for review). Cannabinoids are also believed to mediate the phenomenon of depolarisation-induced suppression of inhibition (DSI; [22–24]). Recently, studies have suggested that CB1R are present at terminals from specific subsets of inhibitory interneurones. For example, fast spiking (FS) inhibitory neurones in neocortex express parvalbumin (PV) but not CB1R, and by contrast, irregular spiking (IS) neurones express CB1R but not PV [25, 26]. Recently, Galaretta et al. [27] have demonstrated that synapses between IS neurones and pyramidal cells express CB1R and show DSI, whereas synapses between FS neurones and pyramidal cells show neither CB1R nor DSI. FS cells are thought to pace fast oscillatory network rhythms such as gamma activity ([28]; and IS cells are thought to possess properties that predispose towards nonrhythmic activity [25, 29]. A subset of neurones that express CB1R but not PV expresses cholecystokinin (CCK), and these neurones have been suggested to act, through DSI, to differentiate subgroups of pyramidal cells into neuronal assemblies which are then entrained by FS cells (“sparse coding” in place cell assemblies, [30]). In this scenario, pyramidal cell activation leads to endocannabinoid synthesis and release, which inhibits IS-cell inputs to the somata and proximal dendrites of active cells, but allows IS-cell-mediated inhibition to remain intact (and ongoing) at less active pyramids. This effect, in turn, allows FS-cells to entrain oscillatory activity only at the disinhibited population of pyramidal cells, effectively selecting that subset for rhythmic activity.

It seems possible that PV−/CCK+/CB1R+ inhibitory interneurones might similarly select populations of pyramidal cells involved in rhythmogenesis in the mEC, which contains both PV+ and PV− neurones [31, 32] and CCK+ interneurones [33], which also express CB1R [34]. We used a selective cannabinoid receptor inverse agonist to globally inhibit CB1Rs during persistent gamma and beta band oscillations in brain slices from the mEC. Under conditions in which CB1 were subject to blockade or inverse agonist effects, we observed a decrease in oscillatory power in gamma and beta bands in layer II. This is consistent with the literature described above [27, 30] and we propose that, in layer II, blockade or inverse agonism of CBRs results in increased irregular phasic inhibition from IS-cells onto pyramidal cells, decreasing the population available to participate in network oscillations and hence reducing field oscillatory power. This appears to be supported by our voltage-clamp recordings showing that LY320135 increased phasic GABAergic inhibition at principal cells in layer II.

When we measured oscillatory activity in layer V, inverse agonists at CBR *increased* gamma and beta power and this appeared to be correlated with decreased superficial beta and gamma power. At first, this appears paradoxical, however, oscillatory activity in specific laminae does not exist in isolation, and we might expect interactions between, as well as within, networks of neurones. Bragin et al. [11] have demonstrated that, in vivo, bilateral ablation of the EC suppresses gamma activity in the dentate gyrus (DG), but augments gamma oscillations in CA3-CA1. As previously discussed, superficial mEC projects to DG, and CA1 projects to deep mEC layers. Given that in our experiments, oscillatory activity in superficial mEC was suppressed, it is reasonable to suggest that this may depress gamma and/or beta activity in DG and enhance such activity in CA3-CA1. This, in turn, would feed through to layer V, where increased gamma and beta power is seen. Hence, although phasic inhibition in layer V appeared to increase in LY320135, it may be that this effect is not involved in selection of neuronal assemblies for oscillatory activity in layer V; rather, excitatory inputs to this area from hippocampus may be the dominant influence on pyramidal cell activity.
